# Inclusion of MCQs written by radiology residents in their annual evaluation: innovative method to enhance resident’s empowerment?

**DOI:** 10.1186/s13244-019-0809-4

**Published:** 2020-01-23

**Authors:** Nadia Amini, Nicolas Michoux, Leticia Warnier, Emilie Malcourant, Emmanuel Coche, Bruno Vande Berg

**Affiliations:** 1 Department of Radiology, IREC, Cliniques Universitaires Saint-Luc UCLouvain, Avenue Hippocrate 10/2942, 1200 Brussels, Belgium; 2 Louvain Learning Lab, ADEF, Grand Rue 54/L1.06.01, 1348 Louvain-la-Neuve, Belgium

**Keywords:** Radiology training, Surveys and questionnaires, Quality, Learning, Internship and residency

## Abstract

**Aim:**

We hypothesized that multiple-choice questions written by radiology residents **(**MCQ^resident^) for their weekly case presentations during radiology staff meetings could be used along with multiple-choice questions written by radiology teachers **(**MCQ^teacher^) for their annual evaluation. The current prospective study aimed at determining the educational characteristics of MCQ^resident^ and at comparing them with those of MCQ^teacher^.

**Methods:**

Fifty-one radiology residents of the first to the fifth year of training took the 2017 exam that contained 58 MCQ^resident^ and 63 of MCQ^teacher^. The difficulty index, the discrimination power, and the distractor’s quality were calculated in the two series of MCQs and were compared by using Student *t* test. Two radiologists classified each MCQ according to Bloom’s taxonomy and frequencies of required skills of both MCQ series were compared.

**Results:**

The mean ± SD difficulty index of MCQ^resident^ was statistically significantly higher than that of MCQ^teacher^ (0.81 ± 0.1 vs 0.64 ± 0.2; *p* < 0.0001). The mean ± SD discrimination index of MCQ^resident^ was statistically significantly higher than that of MCQ^teacher^ (0.34 ± 0.2 vs 0.23 ± 0.2; *p* = 0.0007). The mean number of non-functional distractors per MCQ^resident^ was statistically significantly higher than that per MCQ^teacher^ (1.36 ± 0.9 vs 0.86 ± 0.9; *p* = 0.0031). MCQ^resident^ required recalling skills more frequently than MCQ^teacher^ which required more advanced skills to obtain a correct answer.

**Conclusions:**

Educational characteristics of MCQ^resident^ differ from those of MCQ^teacher^. This study highlights the characteristics to optimize the writing of MCQs by radiology residents.

## Key points

- Scores obtained by PGY1-5 at their annual evaluation increase with education year whoever wrote the MCQs (radiology residents or teachers).
- MCQs written by radiology residents are easier and contain more nonfunctional distractors than MCQs written by radiology teachers, but their discriminant power is higher.
- Memory skills play a more important role in answering MCQs written by residents than by teachers.

## Introduction

Training of radiology residents is generally based on supervised daily clinical practice during scheduled core rotations, autonomous activity during night calls, attendance to multidisciplinary consultations and dedicated radiology-centered lectures, and presentations of clinical cases. Evaluation of their knowledge, skills, and attitudes is an important part of their training and depends on opinions of faculty staff and written or oral exams. In our institution, opinions of faculty staff and MCQ exams are used. Each year, all PG1-5 residents perform a written examination containing 125 single best-option multiple-choice questions addressing knowledge in all fields of medical imaging as outlined in the ESR training curriculum [1].

Multiple-choice question (MCQ) is a validated educational tool for both formative and summative assessment because of its objective output, simplicity of utilization, and informative feedback to exam-takers and teachers [2–6]. For formative MCQ, immediate feed-back to exam-takers promotes reflexion and further learning (catalytic effect). For summative assessment, the MCQ test provides an overall judgment about competence of the exam-taker. In addition, MCQ-based assessment could be more reproducible and objective than exams based on essays or open-ended type questions. There is no indication in the literature that open-questions are more reliable than closed-questions (selected response format) [7, 8].

Writing of MCQs is a difficult task, and teaching staff rarely have the time or incentive to develop high-quality formative questions, focusing instead on material for high-stakes assessments [9, 10]. In the current era of student empowerment, several educational teams proposed to engage students in their education by asking them to submit, review, and discuss MCQs items [3, 9, 11–14]. Collaborative or web-based question-writing is an interesting tool in learning enhancement [9–11, 15–17] because it may stimulate a deeper understanding of the taught subjects and self-monitoring. Although this approach is widely seen among medical students [9, 10, 15–19], there is no evidence in the literature that the same educational approach could be applied to medical residents in training. Therefore, we undertook this prospective pilot study to determine and compare the educational characteristics of MCQs written by radiology residents (MCQ^resident^) and those written by teachers (MCQ^teacher^), combined in a single computer-supported test.

## Material and methods

Since October 2015, radiology residents from the third to the fifth year of residency (PGY3-5) rotating in our academic hospital are asked to integrate two single best-option multiple-choice questions (MCQs) into each of their presentations of clinical cases. MCQ templates including a stem and four-answer options consisting of one correct answer and three distractors were proposed [20–22]. Each MCQ^resident^ was prospectively classified by its author according to eight organs or anatomic systems, three estimated degrees of frequency (frequent, uncommon, rare), and of clinical relevance (important, moderate, less important)*.* The residents were aware that some of their MCQs would be used for their annual evaluation (PGY1-5). In November 2016, 221 resident-authored MCQ^resident^ were available.

In January 2017, a first-year EDIR-certified fellow in radiology with interest in education and the staff member radiologist responsible for the resident’s training (member of the French-speaking National Certification Board for Radiology) have in consensus selected 65 MCQ^resident^, addressing items from all organ systems, with a frequent or an occasional occurrence and with a high or an intermediate degree of clinical relevance. Sixty-five MCQ^teacher^ written by nine radiologists after their lectures were also included in the annual evaluation. The writing of all MCQs was checked for spelling and format (four-option format with only one correct answer) [23]. In February 2017, 51 PGY1-5 residents in radiology took the examination on their PC. Anonymity of the residents was ensured by using pseudos enabling identification of the PGY. All the images contained in the MCQ fulfilled the usual criteria of confidentiality and anonymity for the patients. The participating residents received their personal score immediately at the end of the examination. Correct answers to all MCQs were presented to the residents by faculty staff during two meetings a few weeks after the examination.

### Quantitative analysis

The internal consistency reliability was verified by the Kuder-Richardson formula 20 (KR-20) [24, 25]. Three quantitative parameters including the Difficulty Index, the Discrimination Index, and the number of nonfunctional distractors were calculated for each MCQ [25–29]. The Difficulty Index of an MCQ represents the ratio between the number of students who correctly answered the item and the total number of answering students [26, 27]. A high Difficulty Index (approaching 1.0) indicates an easy question. MCQs were classified as easy (Difficulty Index > 0.70), intermediate (Difficulty Index between 0.70 and 0.30), or difficult (Difficulty Index < 0.30). The ideal value for the degree of difficulty ranges between 0.50 and 0.70 [24]. The Discrimination Index of an MCQ assesses the relationship between how well students did on the item and their total exam score. It is most commonly referred to as the Pearson Point-Biserial correlation (*r*_pbis_) [25, 26]. A high discriminant index indicates that the students who had high exam scores got the item correct, whereas students who had low exam scores got the item incorrect. An ideal range for the discrimination index is above 0.20 [23]. Usual working frame values are as follows: < 0.10 considered of a very poor discrimination power, 0.10–0.20 of a little discrimination power, and > 0.20 of a good discrimination power [26]. Finally, the degree of functionality of the distractors was calculated. A distractor is classified as non-functional distractor (NFD) if less than 5% of students have chosen it [20, 26]. Ideally, there must be no NFD at all, implying the educational power of a question. In our test, there could be 0, 1, 2, or 3 NFD per item. The mean number of NFD was counted per series of MCQs.

### Qualitative analysis

The two radiologists in charge of the evaluation separately classified each MCQ according to Bloom’s cognitive taxonomy that indicates the most probable cognition process needed to correctly answer the item [30]. This hierarchical model of cognitive processes in solving problems includes four levels: remember, understand, apply, and analyze [27]. Based on previously published Blooming Anatomy and Histology tools [31, 32], we used Bloom’s taxonomy type classification system to differentiate among different cognitive levels of radiology MCQs (Table 1).

**Table 1:**
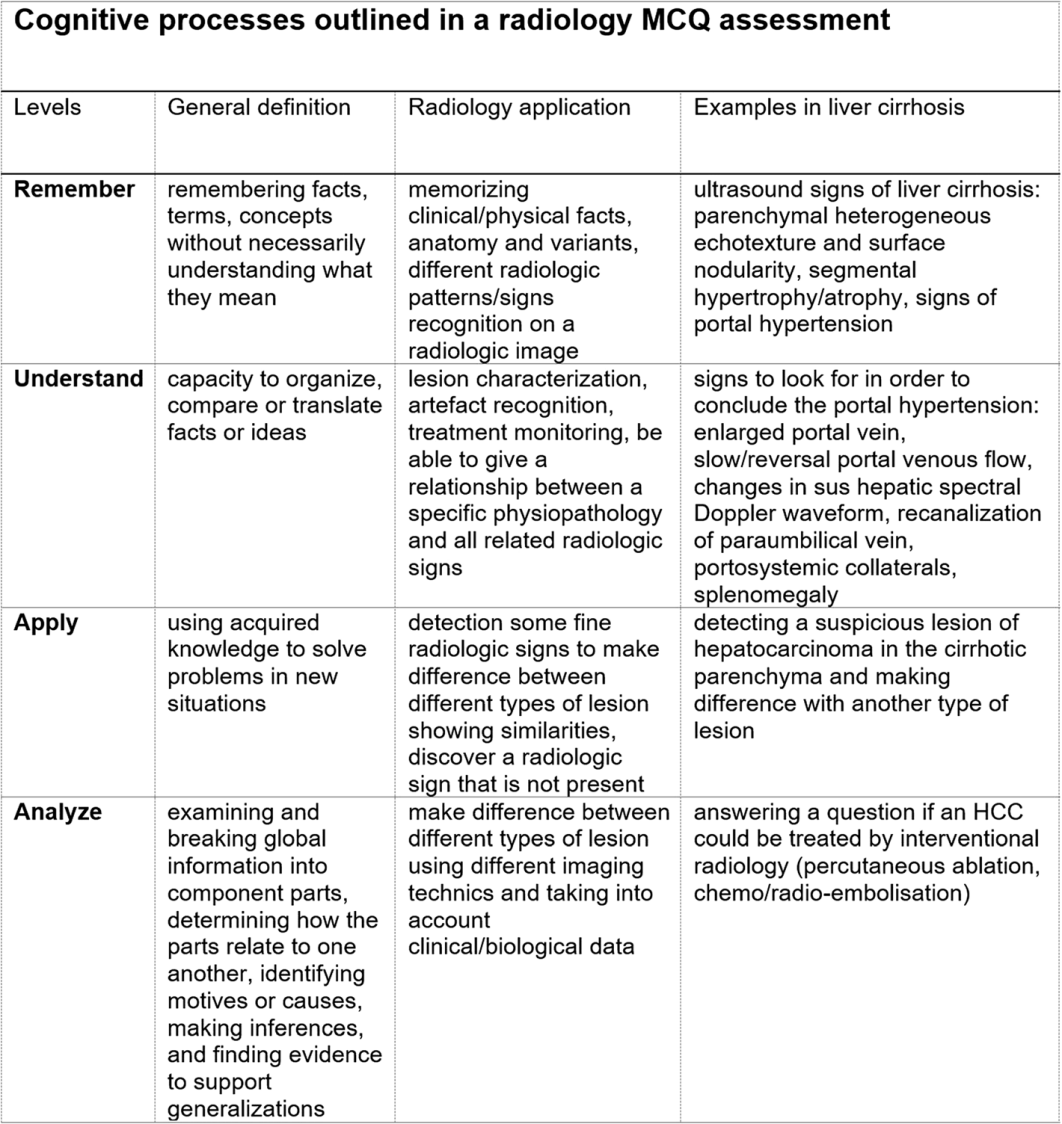
Bloom’s taxonomy type classification system to differentiate among different cognitive levels of radiology MCQs (Bloom’s Taxonomy Radiology Tool)

#### Statistical analysis.

The exam scores of residents were expressed in mean value ± standard deviation (SD) for both questionnaires and were plotted against year of training. Distribution of scores was found to be normal according to the Kolmogorov-Smirnov test. Therefore, a two-sided *t* test (equivalently a Welch test when the equality of variance was not verified according to the *F* test) for independent samples was performed to assess statistical differences between scores from two groups: the MCQ^resident^ and MCQ^teacher^. Due to the multiple comparisons that were performed, a Bonferroni correction of type *p* < 0.05/n^comparisons^ was applied to the tests cited above, and the significance levels were adjusted accordingly.

The mean values ± SD for Difficulty Index, Discrimination Index, and Distractor Functionality of MCQ^resident^ and MCQ^teacher^ were determined. Difficulty and Discrimination Index from residents’ and teachers’ MCQs were compared using the *t* test (after verifying the data distributions normality and the variance equality). The number of non-functional distractors of the two series was compared using the non-parametric Mann-Whitney test (*U* test) and the two-sided Fisher’s exact test with the mid-P approach at *p* < 0.05. The *p* value less than 0.05 was considered to indicate statistically significant difference. The frequency of the highest levels of cognitive process involvement reached by MCQ^resident^ was compared with those of MCQ^teacher^ according to Bloom’s taxonomy [30].

The authorization of our ethical committee was not asked because our study did not involve patients. The project had been validated by resident representatives and faculty staff. All residents were aware of the projects, and they had signed a form for the use of their presentations.

## Results

### Study population

**Fig. 1:**
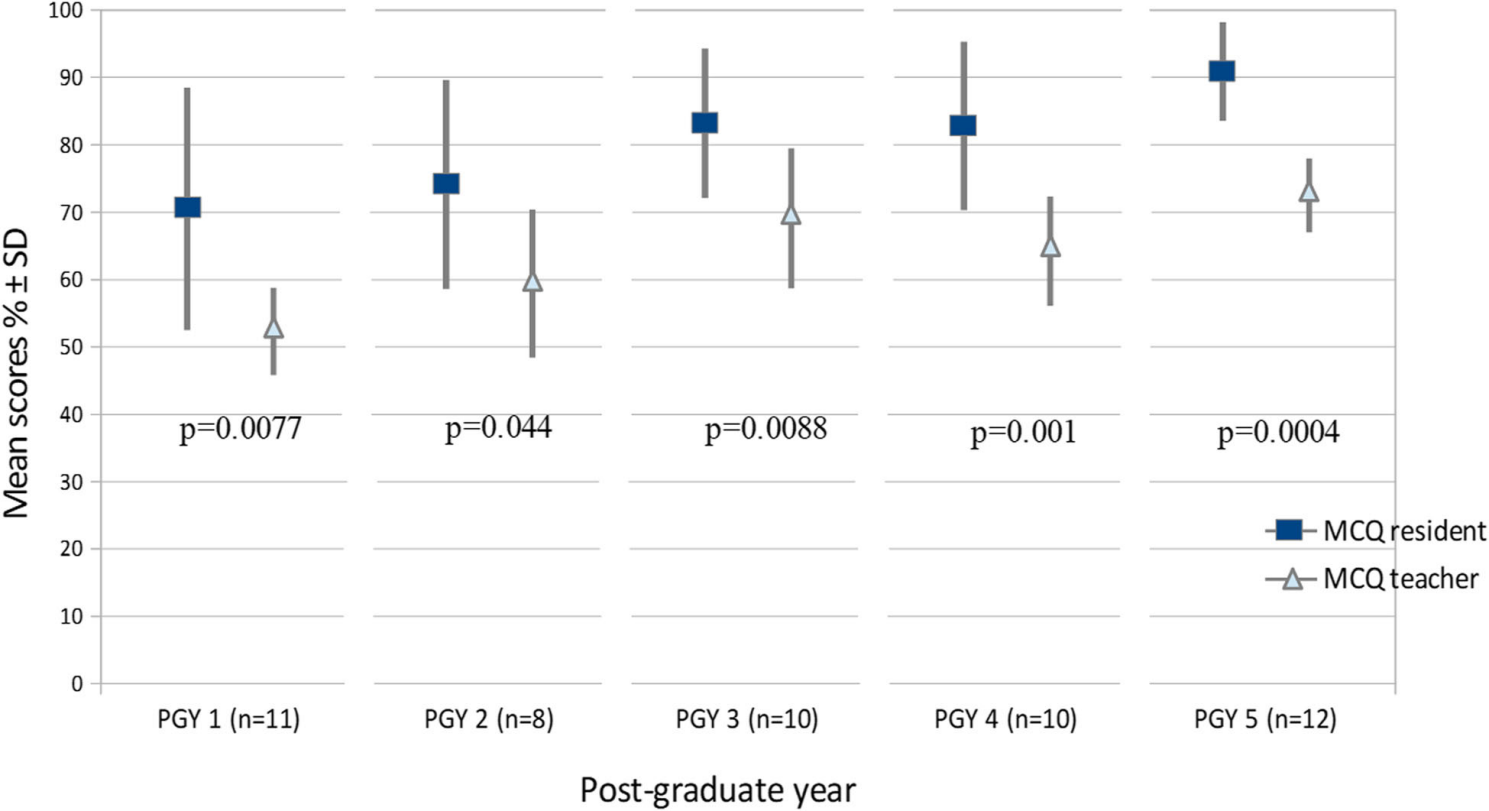
Mean scores of residents for MCQ^resident^ and MCQ^teacher^ per year of residency. A *p* value of less than 0.01 was considered to indicate statistically significant difference

In February 2017, 51 radiology residents (31 men and 20 women, mean age 28 years, range 25–30) from the first to fifth year of training took the exam that initially contained 130 MCQs. Fifty-eight MCQ^resident^ and 63 MCQ^teacher^ were validated and 7 MCQ^resident^ and 2 MCQ^teacher^ were excluded due to technical difficulties during the examination (failure of video on some PCs) (Table 2). Ninety-two out of 121 MCQs included images (57 MCQ^teacher^ and 35 MCQ^resident^). The mean scores (± SD) obtained at the MCQ^resident^ were statistically significantly higher than those at the MCQ^teacher^ for the residents of each year of residency (*p* < 0.01 for each year) (Fig. 1).

**Table 2:**
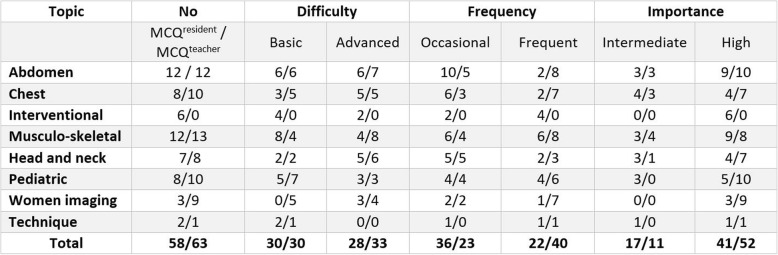
Number of MCQ^resident^ and of MCQ^teacher^ per topic in the 2017 exam according to the degree of difficulty, frequency, and importance as indicated by their authors. Numbers are numbers of items for MCQ^resident^/MCQ^teacher^

#### Quantitative analysis

**Fig. 2:**
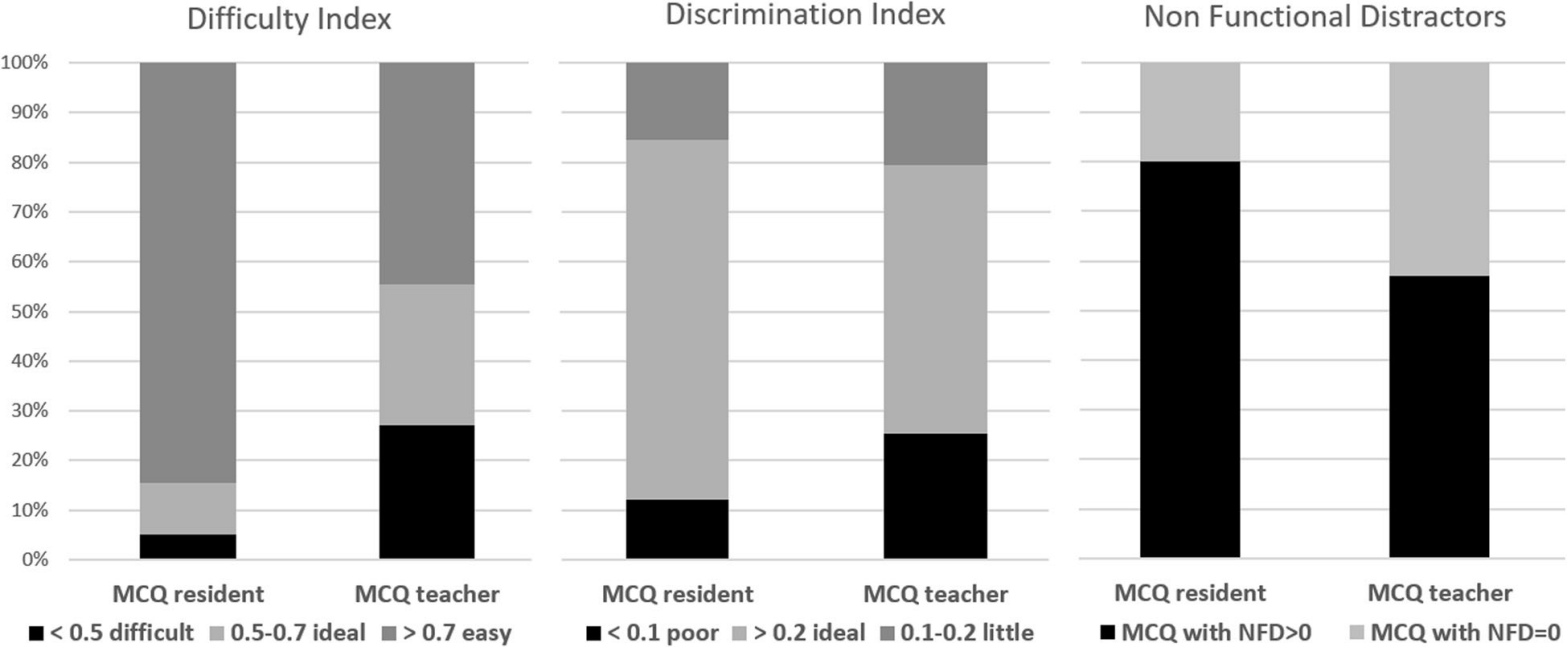
Proportion of degrees of difficulty and of discriminating power and number of NFD in MCQ^resident^ and in MCQ^teacher^

**Fig. 3.**
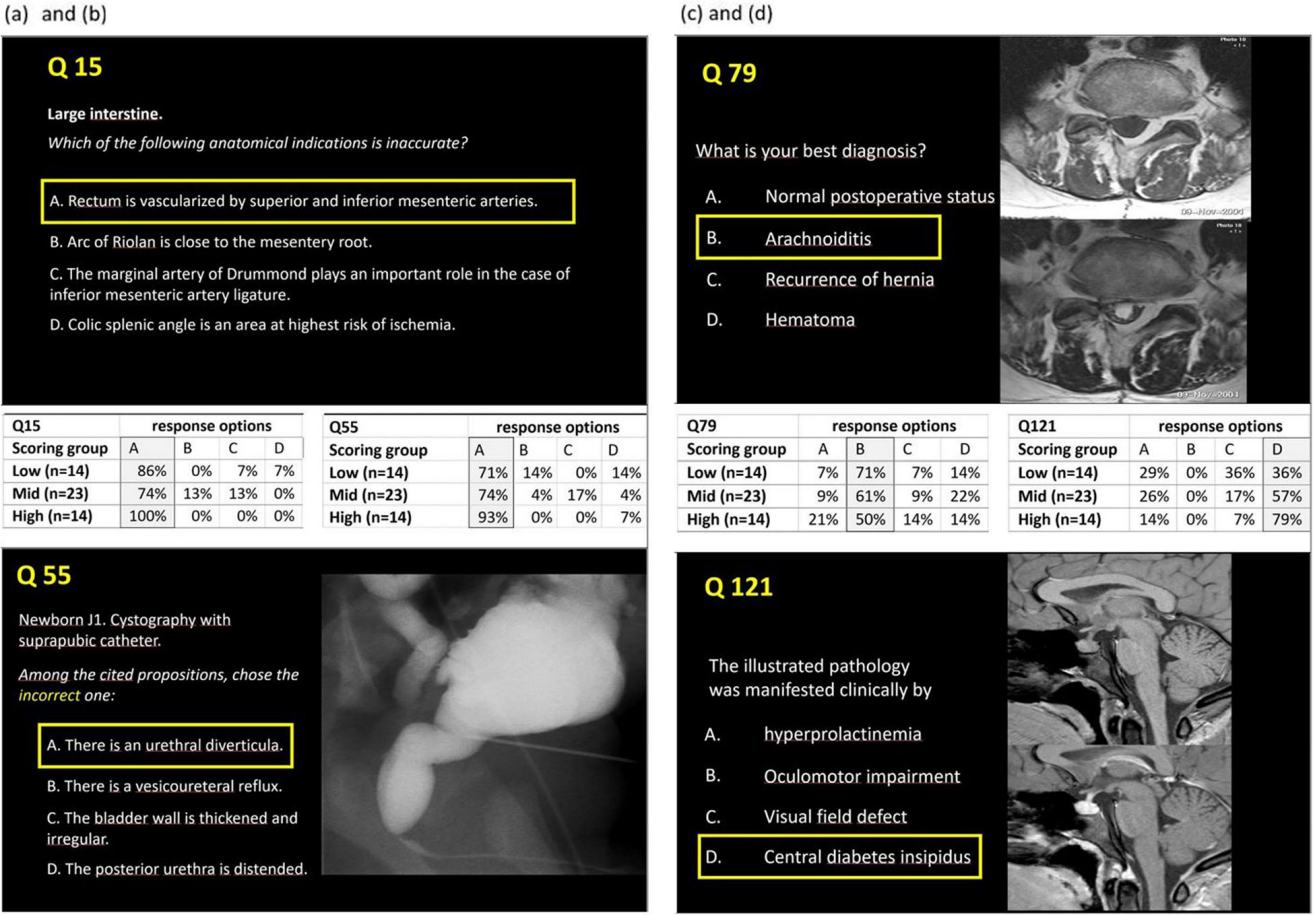
Examples of MCQ^resident^ and of MCQ^teacher^. Correct answer options are highlighted. Percentage indicates the frequency each answer option has been selected by the residents who are classified according to their global performance in this exam. **a** *Q15* (MCQ^resident^ ) was easy (difficulty index = 0.84) and not discriminant (discriminant index = 0.05); it required only recalling skill. **b** *Q55* (MCQ^resident^) had an ideal difficulty index (0.78) and was discriminant (discriminant index = 0,36); correct answer required application/analysis skills. **c** *Q79* **(**MCQ^teacher^) had an ideal difficulty index (0.61) but was not discriminant (− 0,15); correct answer required comprehension/application skills. **d** *Q121* **(**MCQ^teacher^) had an ideal difficulty index (0.57) and was discriminant (0.42); correct answer required analysis skill

The KR-20 value of the test was 0.905. There were less MCQ^resident^ than MCQ^teacher^ with an ideal difficulty index without statistically significant difference (*p* = 0.94), and their mean Difficulty Index (0.81 ± 0.1) was statistically significantly higher than that of MCQ^teacher^ (0.64 ± 0.2) (*p* < 0.0001) (Fig. 2). There were more MCQ^resident^ than MCQ^teacher^ with a good discrimination power (*p* = 0.0002), and the mean Discrimination Index ± SD of MCQ^resident^ (0.34 ± 0.2) was statistically significantly higher than that of MCQ^teacher^ (0.23 ± 0.2) (*p* = 0.0007) (Fig. 2). There were more non-functional distractors in MCQ^resident^ than MCQ^teacher^ (*p* = 0.0022), and the mean number of NFD per MCQ^resident^ (1.36 ± 0.9) was statistically significantly higher than that per MCQ^teacher^ (0.86 ± 0.9) (*p* = 0.0031)(Fig. 2). Examples of MCQ^resident^ and of MCQ^teacher^ with different educational characteristics are given in Fig. 3.

#### Qualitative analysis

The frequency of each cognitive process required for a correct answer is given in Table 3. For MCQ^resident^, recalling-type cognitive process was more frequently required than for MCQ^teacher^ for both reviewers (*p* = 0.004 and 0.001). Application-type (for reviewer 1) and understanding-type (for reviewer 2) cognitive processes were less frequently needed for MCQ^resident^ than for MCQ^teacher^.

**Table 3:**
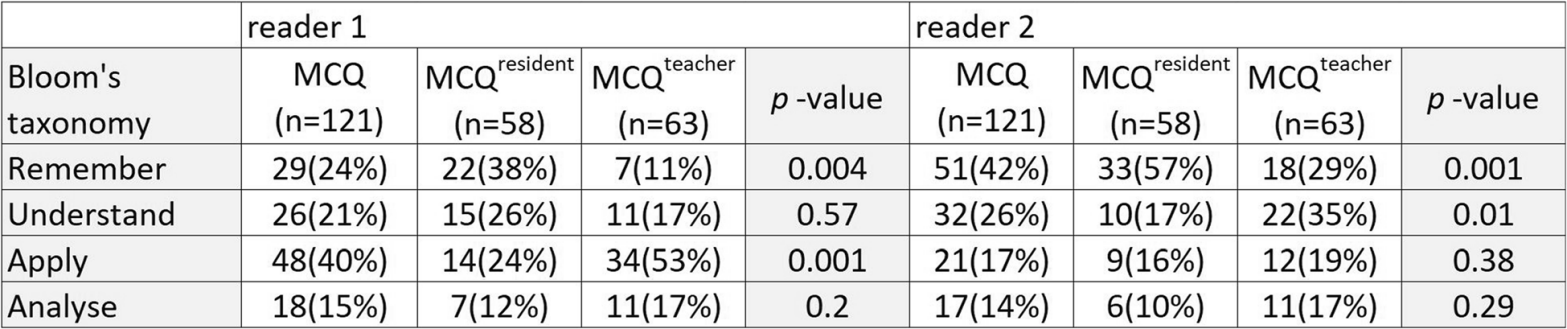
Comparative Bloom’s taxonomy analysis by two readers for MCQ^resident^ and MCQ^teacher^. The *p* value < 0.05 was considered to indicate statistically significant difference

## Discussion

In our institution, the annual evaluation task of the residents consists of three parts: a self-fulfilled logbook (clinical and scientific workload, radiology and multidisciplinary meeting attendance); a summary of the evaluation by the supervising faculty staff of their knowledge, skills, and attitudes; and our MCQ test addressing all radiology subspecialties. The MCQ test is performed on a yearly basis to provide the resident an insight on his/her learning curve throughout the 5 years of residency. The results of the PGY4 and PGY5 MCQs are validated by the National Accreditation Board and integrated in the qualification process, in the absence of a national board examination.

The current study demonstrated that the scores obtained by the residents varied according to their level of training in radiology with a non-exponential improvement throughout the 5 years of residency. Our learning gain curve of radiology residents that seems to decelerate over time was similar to that observed by Ravesloot [28].

Second, the scores obtained in the MCQ^resident^ were statistically significantly higher than in the MCQ^teacher^ for all residents, independently of their post-graduate year. The most likely explanation was that the degree of difficulty of the MCQ^resident^ was lower than that of the MCQ^teacher^ and that the number of NFD was higher in MCQ^resident^ than in MCQ^teacher^. We cannot exclude the hypothesis that the residents deliberately lowered the difficulty level and included NFD because they knew that their MCQs would be used for their annual evaluation. It is most likely that these characteristics are inherent to the degree of qualification of the MCQ writer [33].

Third, the observation that the discrimination index of MCQ^resident^ was higher than that of MCQ^teacher^ warrants further assessment as this feature is important when composing high-quality MCQs. A likely explanation was that MCQ^resident^ were written by PGY3–5 and not by PGY1–2 residents. Therefore, the PGY3–5 residents who, in overall, should obtain the highest scores obtain much better scores than the PGY1–2 residents in the MCQs of their peers than in the MCQ^teacher^, thus artificially increasing the discriminating index of the MCQ^resident^.

Finally, the analysis of the MCQs according to Bloom’s taxonomy demonstrated that the MCQ^resident^ focused more on recalling skills than the MCQ^teacher^ that required the capacity to analyze and apply knowledge. This feature indicates the difficulty in writing high-quality MCQs that require more experience in solving problems [29–31]. However, although Bloom’s taxonomy is a hierarchical model, the lowest levels of the hierarchical Bloom’s taxonomy should not be disregarded as unimportant or unworthy of teaching [34]. Actually, while lesion detection may be considered as a (low) knowledge level (pattern recognition), there is a general agreement on the fact that most errors are detection errors rather than characterization errors. Furthermore, the distinction between the categories can be seen as artificial since any given cognitive task may entail a number of processes. Any attempt to nicely categorize cognitive processes into clean, cut-and-dried classifications undermines the holistic, highly connective and interrelated nature of cognition, a criticism that is directed at taxonomies of mental processes in general [35].

The effects of this collaborative approach for MCQ writing are controversial although it at least contributes to create questions that can support formal or summative evaluations [36]. Aflalo demonstrated the absence of statistically significant improvement in achievements, when comparing the examination grades before and after question generation in a group of 133 students generating questions [37]. Although students were able to write complex MCQs, they found some aspects of the writing process burdensome and tended not to trust the quality of each other’s MCQs [10, 19]. The use of dedicated software like PeerWise which is a freely and globally available online platform allows students to write, share, answer, rate, and discuss peer-written MCQs. Studies demonstrated that PeerWise user students perform significantly better in end-of-course summative assessment than non-user student s[16, 17, 38].

The effect of MCQ format on the resident’s scores was not assessed as questions with videos were eliminated because of technical problems on certain personal computers. While taking the exam, residents were not able to scroll into images. The Clinically Orientated Reasoning Evaluation (CORE) computer-based format that replaced the oral examination in EDIR using DICOM viewer simulating the daily work of radiologists is most likely a better way to evaluate radiology residents [39].

The current study highlighted differences between MCQ^resident^ and MCQ^teacher^ that will be explained to current and future radiology residents in order to increase the quality of their MCQs. In addition, we plan to share this collaborative approach with other training centers to provide a broader supply of MCQ that would decrease the influence on the exam takers.

Our study had several limitations. First, it was a monocenter study with a limited number of MCQs, from residents and from teachers. Second, both MCQs were selected by two radiologists to create a series of MCQs that would cover all fields of diagnostic and interventional radiology. To minimize selection bias, items were selected based on their characteristics indicated by the residents and the teachers and not by reading the MCQs. In addition, questions with a high degree of importance and a frequent occurrence in clinical practice have been privileged. Finally, our results were influenced by the facts that PGY3–5 residents composed the MCQs and that PGY1–5 residents took the examination. Residents were also aware of the fact that their MCQs would be used in the annual evaluation.

In conclusion, the current study demonstrated that the educational characteristics of MCQ^resident^ differ from those of the MCQ^teacher^ in many ways. The clear identification of these differences enabled us to indicate points of attention to address in MCQ writing guidance in order to achieve higher quality examinations with the collaboration of the teaching staff.

## Data Availability

The datasets used and/or analyzed during the current study are available from the corresponding author on reasonable request.

## Abbreviations

CORE: Clinically Orientated Reasoning Evaluation
DICOM: Digital Imaging and Communications in Medicine
EDiR: European Diploma in Radiology
ESR: European Society of Radiology
MCQ: Multiple-choice question
MCQ^resident^: Multiple-choice question written by residents
MCQ^teacher^: Multiple choice-question written by teachers
NFD: Non-functional distractor
PGY: Post-graduate year
SD: Standard deviation
